# Hospital Surgeons of Paris

**Published:** 1830

**Authors:** 


27. *Hospital Surgeons of Paris.* We are indebted to the Boston journal for a sketch of some of the principal hospital surgeons of Paris; an extract of which, we fancy, will not be without its interest, to those who are accustomed to contemplate these distinguished men only through their writings. The writer is a Yankee student of medinine, at present pursuing his studies in the French metropolis:

At the Hotel Dieu, Dupuytren begins his visit at daylight, after which he gives a lecture, and has operations. He is an admirable lecturer, and has the art of making the most trifling subject interesting. He is a man about 55, I should think, or upwards, strong muscular figure, with a fine intelligent countenance, and with grey hair. At the hospital, he always wears a white apron of coarse cloth. The principal operation I have seen him perform, was for the stone, upon a child about three years old. He introduced a full-sized sound, with a large groove, and having a large probe extremity, into the bladder, and then, with a sharp-pointed bistoury, he made a transverse semilunar incision about three lines above the margin of the anus. He then introduced the concealed gorget, and afterwards a pair of forceps, with which he immediately withdrew a stone of large size. The whole operation was performed in an inconceivably short space of time; the child appeared to suffer very little during or after the operation, and was perfectly well in a few days. It was the third operation he had undergone.—Rather a singular operation Dupuytren performed the other day, was trepanning the os humeri below the insertion of the deltoid muscle, for disease of the bone. Dupuytren, in his visits, uses the most endearing epithets, as *ma belle fille, mon bon garcon*, etc., but he is excessively irritable, and if they do not answer quick enough, or give unsatisfactory answers, he abuses them at a great rate.

At La Pitie is Lisfranc, the rival perhaps of Dupuytren, or second only to him in operative surgery. He is a much younger man than the former, tall, handsome, and of a more commanding figure, with an expression slightly sarcastic. In addition to the apron, he visits and lectures in a black night-cap. He is much admired as a lecturer;—his hours are the same with those of Dupuytren, with whom he is at sword-points, and whom he does not spare in his lectures. As I have attended the lectures of the former, I have heard him only once or twice;—I saw him cut off a leg, which he did with wonderful dexterity and expedition. The rapidity with which they all operate, is the most remarkable feature I have observed in the French Surgery. It is perhaps owing to the facility of constantly pursuing dissections here, to their devoting their lives to one branch of the profession, and to the number of operations they are called to perform: whether, after all, it is consistent with the safety of their patients, is a thing which I greatly doubt.

At the Hospital Neckar, Civiale has generally two or three patients upon whom he operates, every Saturday, for extraction of the stone through the urethra. He is a young man, I should think about thirty-five, has a very pleasant intelligent countenance, and appears to be a perfect gentleman. He seems to be the particular favorite of the Americans, and is followed almost exclusively by an American class.

From the Hospital Neckar, we usually go to La Charite, where Boyer and Roux operate. Boyer looks like anything but a great man. He is a fat square figure, and in his old coat and bloody apron, might be taken for a hogkiller. He operates most generally for fistulæ in ano. He appears to possess a great deal of humor, is very fond of gossiping with the students, generally sits a long time talking with them after the visit, and will keep the class laughing for an hour, in a lecture on the fistulæ.—Roux is about thirty: he is said to perform many operations with great dexterity; I have seen him only once. He then performed the operation for brachial aneurism. He put a roller of linen between the artery and the ligature, in the manner recommended by Boyer. Boyer, by the way, has lately performed an important operation,—the extermination of a large portion of the rectum,—an account of which you have probably seen by this time.

At the School of Medicine, Orfila is the most popular lecturer. The doors are opened half an hour previous to the commencement of the lecture, and, half an hour before this they are surrounded by hundreds of students in defiance of the cold At last the doors are opened, and the crowd rush forward with all their might, overthrowing and trampling down some, and squeezing the breath almost out of the body of others. I attended his lectures at first, when the throng was not so great, and wished much to continue, but I found it impossible to get a place near enuogh to hear him. He is a very animated lecturer, and speaks with great rapidity. His lectures on the application of Chemistry to Medicine and the Arts, are among the most useful here.

